# Effect of Fibres on the Failure Mechanism of Composite Tubes under Low-Velocity Impact

**DOI:** 10.3390/ma13184143

**Published:** 2020-09-17

**Authors:** Jie Xiao, Han Shi, Lei Tao, Liangliang Qi, Wei Min, Hui Zhang, Muhuo Yu, Zeyu Sun

**Affiliations:** 1 College of Materials Science and Engineering, Donghua University, Shanghai 201620, China; jiex0616@dhu.edu.cn (J.X.); dhuemc@126.com (H.S.); taoleitim@163.com (L.T.); 18019362276@163.com (L.Q.); wmindhu@163.com (W.M.); zhanghui@dhu.edu.cn (H.Z.); yumuhuo@dhu.edu.cn (M.Y.); 2 Shanghai Collaborative Innovation Center for High Performance Fiber Composites, Donghua University, Shanghai 201620, China; 3 Research Center for Analysis and Measurement, Donghua University, Shanghai 201620, China; 4 Shanghai Key Laboratory of Lightweight Structural Composites, Shanghai 201620, China

**Keywords:** composite tubes, fibres, low-velocity impact, failure mechanism, radial residual compression strength

## Abstract

Filament-wound composite tubular structures are frequently used in transmission systems, pressure vessels, and sports equipment. In this study, the failure mechanism of composite tubes reinforced with different fibres under low-velocity impact (LVI) and the radial residual compression performance of the impacted composite tubes were investigated. Four fibres, including carbon fiber-T800, carbon fiber-T700, basalt fibre, and glass fibre, were used to fabricate the composite tubes by the winding process. The internal matrix/fibre interface of the composite tubes before the LVI and their failure mechanism after the LVI were investigated by scanning electric microscopy and X-ray micro-computed tomography, respectively. The results showed that the composite tubes mainly fractured through the delamination and fibre breakage damage under the impact of 15 J energy. Delamination and localized fibre breakage occur in the glass fibre-reinforced composite (GFRP) and basalt fibre-reinforced composite (BFRP) tubes when subjected to LVI. While fibre breakage damage occurs globally in the carbon fibre-reinforced composite (CFRP) tubes. The GFRP tube showed the best impact resistance among all the tubes investigated. The basalt fibre-reinforced composite (BFRP) tube exhibited the lowest structural impact resistance. The impact resistance of the CFRP-T700 and CFRP-T800 tube differed slightly. The radial residual compression strength (R-RCS) of the BFRP tube is not sensitive to the impact, while that of the GFRP tube is shown to be highly sensitive to the impact.

## 1. Introduction

Composite materials are widely applied in many fields such as aerospace, shipping, rail traffic, automobiles, and construction owing to their light weight, corrosion resistance, fatigue durability, and excellent resistance to high and low temperatures. Carbon fibre, glass fibre, and basalt fibre are the main types of fibres that exhibit the best performance while processing composite materials, which differ considerably based on performance and applications. A carbon fibre exhibits the best specific strength and modulus, compared with the other two types of fibres. Carbon fibre-reinforced composites (CFRPs) have been applied in the aerospace, military defence, and civil industries [1,2]. Basalt fibres not only exhibit moderate mechanical properties, but are also highly cost-effective, and basalt fibre-reinforced composites (BFRPs) are widely used in construction and manufacturing. Glass fibre-reinforced composites (GFRPs) find various applications in the fields of electronics, transportation, and construction owing to the good machining performance and high-strain-failure coefficient of glass fibres [3,4,5].

With the increasing need to reduce emissions, energy saving and eco-friendly materials such as lightweight composite tubular structures have emerged to have major applications in transmission systems, pressure vessels, sports equipment, and hydrogen cylinders [6,7]. When unavoidable low-velocity impact (LVI) accidents such as the impact of a tool drop or of flying debris occur during production, maintenance, or repair, they can cause barely visible impact damage (BVID) in the form of matrix cracking, delamination, and fibre breakage/rupture. This is because of the poor impact resistance of the composites under impact, which is the energy required to cause damage. This BVID can significantly reduce the residual mechanical strength and structural integrity of the composites, thereby affecting the normal operation of the system and sometimes even leads to unforeseen losses [8,9,10].

In recent years, research on the impact behaviour of composite laminates has been increasing, and the researchers have come to a consensus regarding the damage mechanism and failure mode. Wang et al. [11] observed two different tensile failure modes in composite laminates subjected to various impact energies. They proposed that the degradation process of the residual tensile strengths of the laminates could be divided into three stages depending upon the different impact energy; the degradation amplitude is affected by the stacking sequence. Sarasini et al. [12] studied the LVI behaviour of hybrid laminates manufactured via resin transfer moulding. The results indicated that the intercalating configuration of the hybrid laminates showed better impact energy absorption capability and enhanced impact resistance with respect to the all-aramid laminates. H. Debski and P. Rozylo [13] studied the effect of LVI damage location on the stability and post-critical state of composite columns under compression. Moreover, they build a model of LVI damage of composite plates subjected to Compression-After-Impact (CAI) testing [14]. On the contrary, process conditions of composite tubular structures are uncontrollable because of pressurisation, high porosity, and high fluctuation in product performance. There were certain difficulties encountered while attempting to reach a consensus on the damage mechanism and failure mode of the composite tubes after LVI process. Gning et al. [15] investigated the transverse impact behaviour of glass fibre reinforced epoxy resin matrix composite cylinder with a thick wall; they found that delamination initiates at the impact energy of 4 J and it further propagates with the increase in impact energy. Phadnis et al. [16] found that the integration of glass fibre in the laminate improved the impact performance because of the high fracture strain of the glass fibre, which hindered the propagation of damage in the inner layer. Kara et al. [17] studied the impact behaviours of filament-wound CFRP composite tubes at cryogenic temperatures. It was observed that the damages of the specimens increased as the temperature decreased for all test samples. Kim et al. [18] investigated the LVI impact resistance of aluminium/composite hybrid tubes. They suggested an optimal plying sequence for the composite material and an optimal aluminium tube thickness. Liu et al. [7] studied the behaviour and damage of CFRP tubes under transverse LVI. The results showed that circumferential cracks generated easily in the tubes at the low impact energies of 5 and 10 J. Finite element methods (FEM) has more advantages in predicting the damage behaviour and failure mechanism of composite tubes from the aspects of crush trigger, design variables and loading conditions. There has been more and more research focused on the impact behaviour of composite structures by combing with experimental and numerical methods. Wu et al. [19] studied the transverse low-velocity impact response and residual compression behaviour of braided composite tubes with different ply numbers by experimental and numerical methods. A two-step finite element model was established to reveal damage mechanisms of the tubes under impact loading. They found that the wall thickness had a significant influence on the impact response and obvious structural deformation occurred in the 2-ply tube when subjected to impact loading, resulting in a large projected delamination area. Alam et al. [20] performed a finite element (FE) simulation of CFRP-strengthened square hollow section steel columns subjected to transverse impact to predict their behaviour and failure mechanism. The results showed that the strengthening method improved the impact resistance by reducing the lateral displacement of the strengthened column by approximately 58% compared with that of the bare steel column.

Owing to the particularity of the structure of the composite tubes, the factors affecting their impact resistance, including the material properties of the reinforced material, matrix material, structural properties of the layup mode, and tube size, are also very complex. These have not been sufficiently studied thus far. Mokhtar et al. [21] proposed that the maximum damage diameter of the basalt composite pipes increases significantly with the increase in the winding angle and impact energy, and the shape of the damaged area changes accordingly. The basalt composite pipes with a large winding angle absorb less energy. Liu et al. [7] reported that tubes with mixed ply angles exhibit better impact resistance in terms of energy absorption. Hassan et al. [22] proposed the study of LVI damage of woven fabric composites for different specimen thicknesses via finite element simulation and experimental verification. The simulation and experimental results were in agreement in terms of maximum contact force and time.

The above studies mainly focused on the impact response of composite tubes or metal/composite tubes to the environmental factors (temperature, humidity, and heat, etc.), impact energy, structural parameters, and other conditions. The effect of material properties (especially the reinforced fibre of the composites), on the impact performance and even the failure mechanism during the LVI have been investigated in few studies. Moreover, most of the residual mechanical strength after impact damage is based on the bending and torsional properties of the tubes; the radial residual compression strength (R-RCS) of the composite tubular structures, which is essential for pressure tubes, has rarely been reported. Herein, LVI damage responses and R-RCS of the four types of fibre (including carbon fibre-T800, carbon fibre-T700, basalt fibre and glass fibre) reinforced epoxy resin matrix composite tubes are investigated and discussed; computed tomography (CT) and scanning electric microscopy (SEM) are utilised to understand the inner damage and failure mechanism. This work aims to compare the impact behaviours of different fibre-reinforced composite tubes and understand how fibres affect the failure mechanisms of composite tubular structures. This may indirectly help in choosing and designing the materials to improve the anti-impact performance of composite tubular structures.

## 2. Materials and Methods

### 2.1. Materials

The reinforcement materials used in this study were carbon fibre (T700SC-12000-50C and T800HB-12000-50B, Toray Industries, Tokyo, Japan), basalt fibre (BCR13-198-2400-101T26-2W, Shijin Basalt Fiber co. LTD, Dongyang, China), and glass fibre (ECT550E-1200, PPG Industries Inc., Pittsburgh, PA, USA). Epoxy resin (Bisphenol A epoxy, Wuxi Phoenix Resin Company, Wuxi, China) was used as a matrix material, and the curing agent was 1-(cyanoethyl) Imidazole (Sinopharm Chemical Reagent Co., Ltd., Shanghai, China). The composite tubes were prepared using one-step wet winding technology in a four-axis winding machine (CRJ-12, LONGTEC, Xi’an, China).

### 2.2. Fabrication of Composite Tubes by Wet-Filament Winding

**Figure 1:**
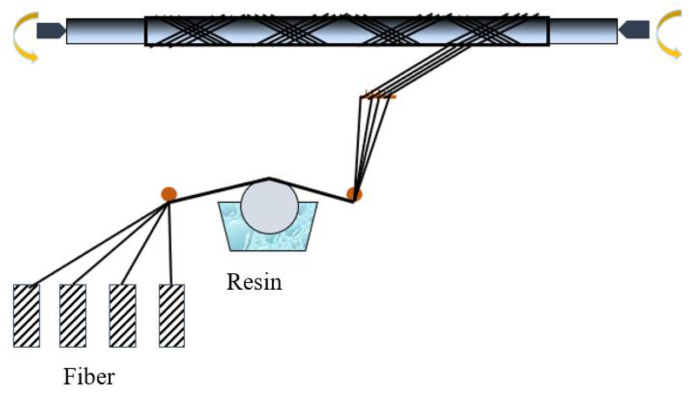
Schematic of the wet-filament winding process.

The wet-filament winding method is generally used to fabricate symmetric structures with rotating bodies and positive curvatures. The process flow diagram is shown in Figure 1.

In this study, carbon fibre-T800, carbon fibre-T700, basalt fibre and glass fibre are the four types of fibres that were each wound with epoxy resin to fabricate a total of four composite tubes with a winding angle of [±25_4_/90_2_]. The mechanical properties of the four types of fibres are listed in Table 1.

The material parameters of the FRP materials are listed in Table 2. The material parameters including Young’s modulus, shear modulus, and Poisson’s ratio have been measured according to GB/T 1458-2008.

**Figure 2:**
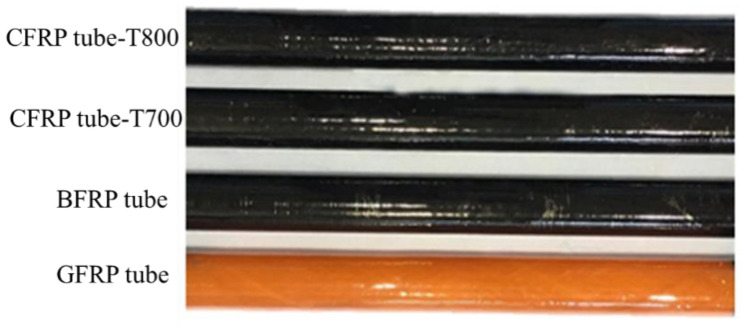
Composite specimens reinforced by four types of fibres.

The composite tubes with a length of 1200 mm were prepared by filament winding processing, as shown in Figure 2. A cutting machine was applied to cut the tubes into short tubes of 100 mm length, 70 mm inner diameter, and about 3.0 mm thickness.

**Figure 3:**
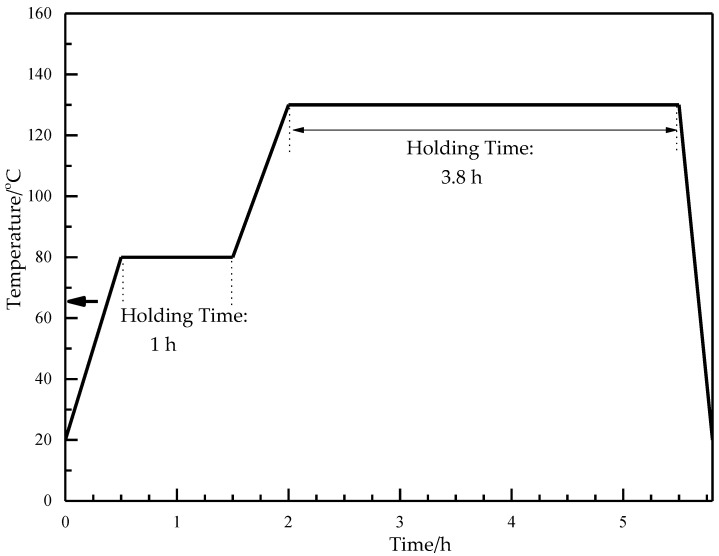
Subsection heating scheme of the moulding process.

The curing stage of the resin system played an important role in the moulding process. The subsection heating scheme shown in Figure 3 was set as the optimal heating solution.

### 2.3. Low-Velocity Impact Tests

**Figure 4:**
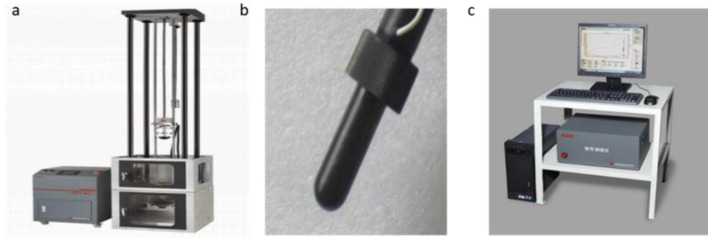
(**a**) Low-velocity impact test set-up; (**b**) impactor; (**c**) signal receiver.

To simulate the LVI process on the composite tubes, the MTS ZCJ1302-AD drop hammer tester (MTS Industrial Systems (China) Co., LTD, Shenzhen, China) was used to affect the specimens (Figure 4). The impactor had a hemispherical tip with a mass of 2 kg and a diameter of 12.5 mm. A PCB Quartz ICP force sensor (PCB Piezotronics, Inc.Buffalo, New York, NY, USA) was used to measure the force-time responses (in terms of the test time, impact force, impactor velocity, displacement, and energy) of the samples. The impactor was dropped from a certain height to achieve the impact energy level of 15 J by striking once only.

**Figure 5:**
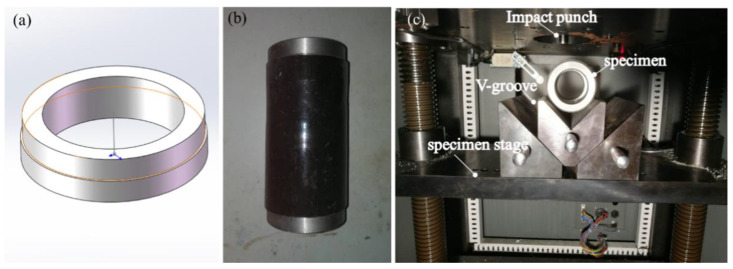
(**a**) Geometry of metal kit; (**b**) composite tube-installed metal kits (**c**) actual low-velocity impact (LVI) tests.

During the experiment, large deformation was observed in the short tubes subjected to impact, and layer overflow also occurred easily. This contributed to the lack of restraints at both ends. However, this does not agree with the actual working conditions. To address this problem, metal kits (Figure 5a) were installed on both ends of the tubes as shown in Figure 5b. During the LVI tests, composite tubes were placed on the V-groove to restrain the slippage of the tubes and improve the accuracy, as shown in Figure 5c.

### 2.4. Damage Characterization

An X-ray micro-CT system (GE, phoenix v|tome|x m, GE Sensing & Inspection Technologies, Cincinnati, OH, USA) was employed to characterise the internal damages of the composite tube after the LVI and R-RCS tests. The tomography system equipped with a 300 kV micro focus X-ray tube and a highly dynamic DXR digital detector array provided detailed three-dimensional information about the specimens.

### 2.5. Radial Residual Compression Strength Test

**Figure 6:**
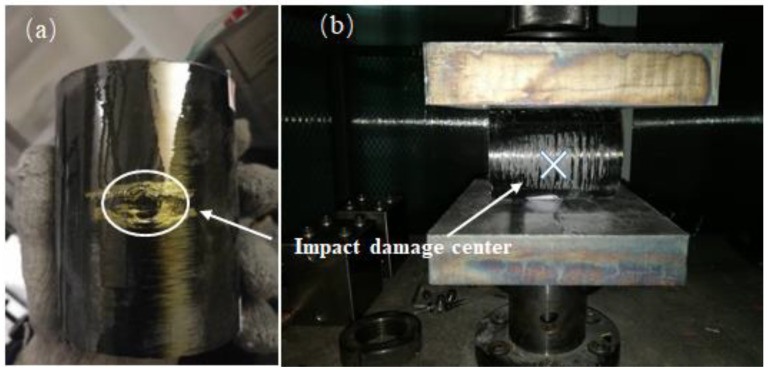
Radial compression test: (**a**) impact-damaged composite tube; (**b**) location of damaged tube.

R-RCS tests were conducted to study the radial compressive strength loss of the composite tubes tested on the LD23 universal testing machine (Shenzhen Rambo Sansi Material Testing Co. LTD, Shenzhen, China). A constant displacement rate of 10 mm/min was employed and the tests were carried out according to GBT 5352-2005 standard. As shown in Figure 6**,** the composite tubes underwent radial compression between two parallel load plates with the dimensions of 200 mm × 150 mm × 30 mm. The impact position was located along the largest transverse size. The experiment was stopped when the deformation rate of the diameter reached 60% or the structure was clearly damaged. The deformation rate *P* was calculated using the following equation:

(1) $$P\left(\%\right)=\frac{∆Y}{d}\times 100$$

where ∆*Y* is the change in the variation value of the diameter and *d* is the mean inner diameter of the composite tube.

### 2.6. Scanning Electron Microscopy (SEM) Characterization

The SEM analysis of the tubes was carried out on a Hitachi SU8010 microscope (Hitachi, Tokyo, Japan) to observe their internal matrix/fibre interface and the dispersion of the fibres in the resins after breaking off in liquid nitrogen.

## 3. Results and Discussion

### 3.1. Low-Velocity Impact Response

**Figure 7:**
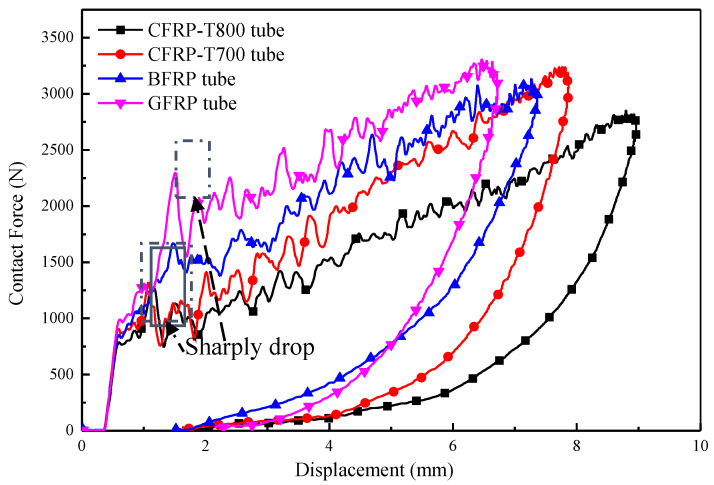
Force–displacement (F–D) diagrams for the four types of fibre-reinforced composite tubes.

The diagrams for contact force, displacement, impactor velocity, and absorbed energy were studied to assess the impact behaviour of the four types of fibre-reinforced composite tubes subjected to LVI. Displacement is obtained by recording the vertical displacement of the impactor nose. Figure 7 exhibits four closed contact force–displacement (F–D) curves, indicating that the impacted tube is not perforated; an open curve is observed in the presence of perforation. Small oscillations in the rising phase can be observed on the force-time curve in Figure 7 due to localized damage induced by the impactor, which indicates that the composite tubes are constantly damaged. The contact force near the value of 1250 N drops sharply at CFRP-T700 and CFRP-T800 tubes. The contact force at the GFRP tube decreases violently under about 2250 N, which may be caused by serious damage. Compared with the other three tubes, the contact force at BFRP tube is relatively stable, of which no big fluctuations appear during the whole process. During the loading phase of impact, all curves exhibit the same trend. The area under the curve is the deformation energy that is initially progressively transferred from the impactor to the tubes; subsequently, the deformation energy is returned from the tubes to the rebounding impactor. The area inside the loop refers to the energy absorbed during the impact. After the tubes absorbed certain impact energy, the damage mechanism was activated, and then a fluctuant rising of the force appears. As displayed in Figure 7, during the impact and the rebound process, the contact force values of the FRP tubes at the same displacement show the following trend: GFRP > BFRP > CFRP-T700 > CFRP-T800.

**Figure 8:**
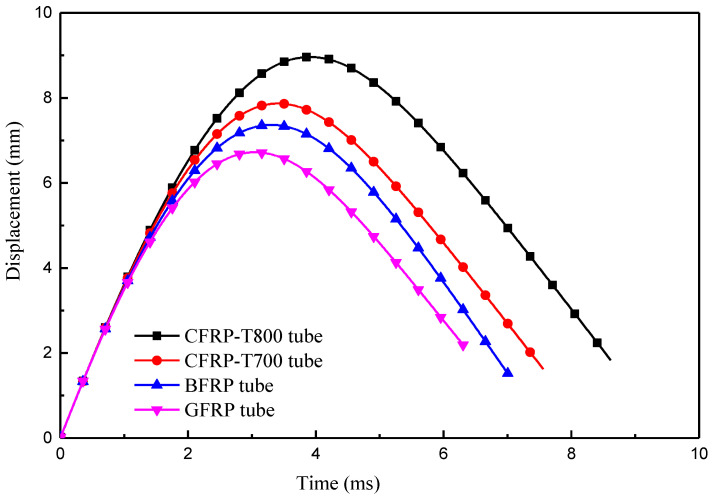
Displacement–time (D–T) diagrams for four types of fibre-reinforced composite tubes.

Figure 8 illustrates the displacement–time (D–T) diagram of the four types of fibre-reinforced composite tubes. The maximum displacements of CFRP-T800, CFRP-T700, BFRP, and GFRP tubes are 8.96 mm, 7.87 mm, 7.36 mm, and 6.72 mm, respectively. The residual deformation is the irreversible deformation caused after impact and is the result of damage to the tubes. The residual deformation values of the four tubes were 1.83 mm, 1.62 mm, 1.47 mm and 2.15 mm, respectively. The ratio of residual deformation to the maximum deformation is considered to be the standard of measurement of the anti-impact deformability of the composite tubes. The ratios of the four tubes are 20.4%, 20.5%, 20.0% and 32.0%, respectively.

### 3.2. Low-Velocity Impact Damage Characterisation

**Figure 9:**
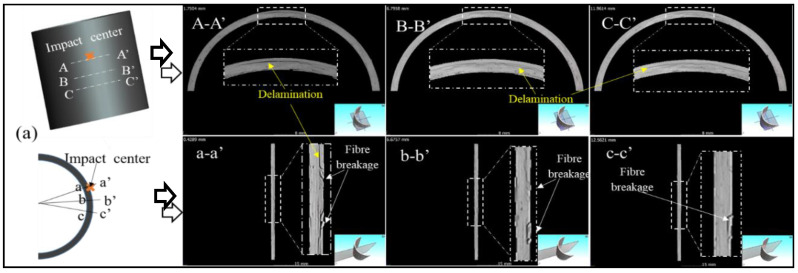

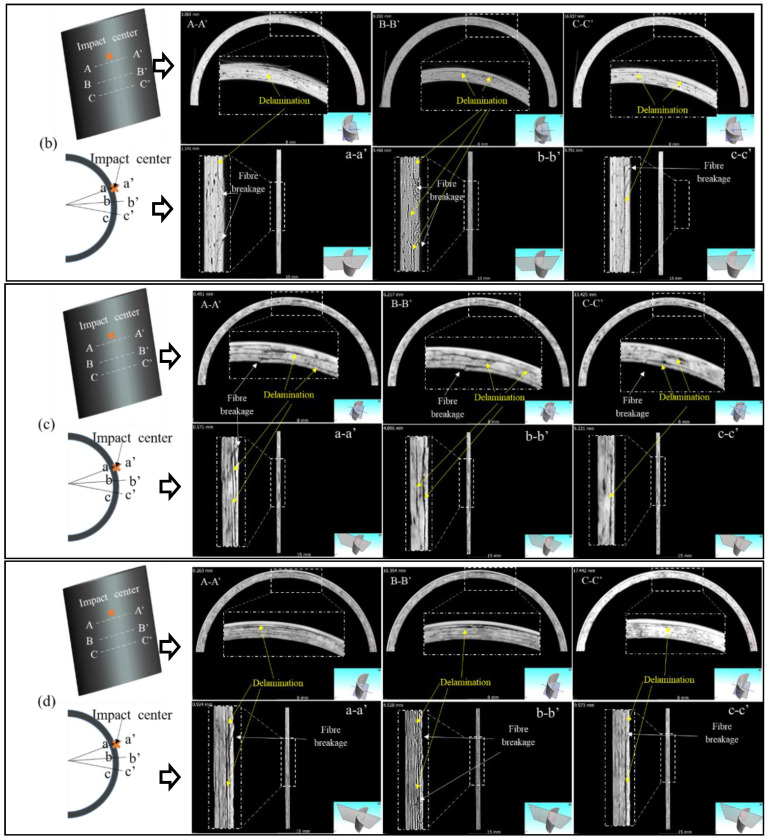
Tomographic cross-sectional views for four types of fibre-reinforced composite tubes after impact at different positions: (**a**) CFRP-T800 tube, (**b**) CFRP-T700 tube, (**c**) BFRP tube and (**d**) GFRP tube.

To demonstrate the above failure behaviour and to better understand the failure mechanisms after impact, especially when the damage is underneath the material surface and cannot be seen by the naked eye, CT images of specimens can be utilised. Three circumferential-sectional views (A-A’, B-B’ and C-C’) and three axial-sectional views (a-a’, b-b’ and c-c’) are chosen from the centre to the edge of the impact location in the tubes, as displayed in Figure 9.

Figure 9 displays the CT images of composite tubes after impact, wherein, images (a) to (d) is for CFRP-T800, CFRP-T700, BFRP and GFRP tubes, respectively. From CT images, more types of damage mechanisms can be observed in greater details, including fibre breakage, fibre rupture and delamination [23]. Figure 9a illustrates the failure mechanism of CFRP-T800 tube after impact. The degree of the damage weakens along with the distance from the impact centre to the edge. Figure 9 (b) displays the failure mechanism of CFRP-T700 tube after impact. Similarly to the CFRP-T800 tube, obvious delamination damage is observed and the global fibre breakage occurs around the impact centre. This is related to the lower elongation at break and the greater brittleness of the carbon fibres. During the LVI process, the contact force of both CFRP-T800 and CFRP-T700 tubes drops sharply when it reaches 1300 N (Figure 7), which may be related to the global fibre breakage damage, as seen in Figure 9a,b. It is clear that the impact behaviour of the two composite tubes is similar at 15 J energy. Figure 9c displays the failure mechanism of the BFRP tube under LVI. Differing from the CFRP tubes, the failure mechanism of the BFRP tube is mainly in the form of delamination, while the fibre breakage is observed locally. Special attention should be paid to the fact that the fibre breakage damage of the BFRP tube occurs in the impact centre, which is different to the CFRP tubes, indicating that the fibre has an influence on the FRP tubes when subjected to LVI. Figure 9d displays the failure mechanism of the GFRP tube after impact. Similarly to the BFRP tube, the delamination damage is also the main failure mechanism and localized fibre breakage is observed. From the above, it can be seen that, when subjected to the impact energy of 15 J, fibre breakage and delamination are the main forms of damage in the FRP tubes, while fibre rupture is rarely seen, which requires a large amount of energy [24]. The interfacial adhesion between fibres and resins differ a lot, as well as the mechanical properties of composite materials reinforced by different fibres. This results in the damage degree of composite tubes when subjected to LVI.

**Figure 10:**
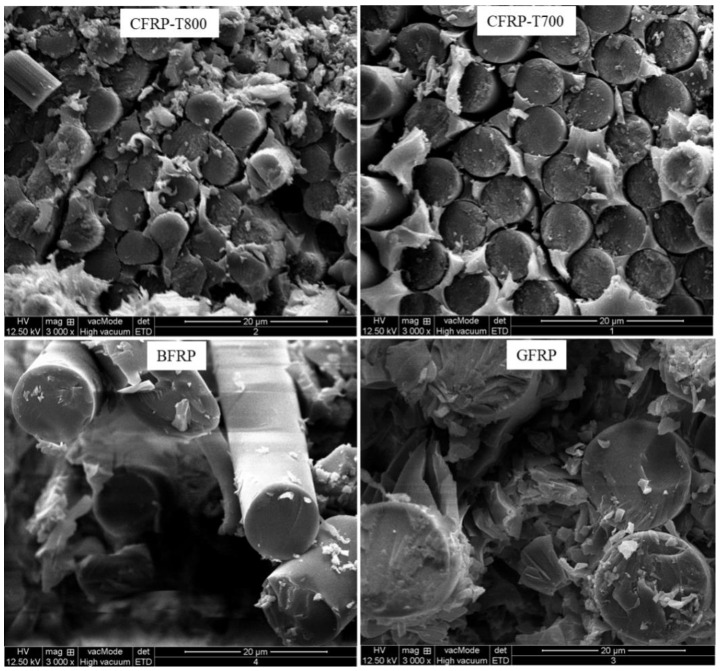
Sectional SEM images of the un-impacted FRP composite tubes reinforced by the four types of fibres.

The mechanical properties of the composite structures play an important role in the impact behaviour of composite tubular structures. The matrix/fibre interfacial properties of the composite structures play an important role in the structural impact resistance. To examine the internal matrix/fibre interface of the un-impacted reinforced tubes, their SEM images were obtained (Figure 10). It can be clearly observed from the SEM images that most of the basalt fibres were pulled out from the resins and a small amount of resin adhered to the pulled-out fibres, indicating that a weak interfacial adhesion existed between the fibres and resin in the case of the BFRP tubes. It could be used to explain the failure mechanism of the BFRP tubes when subjected to impact. Furthermore, the relatively low mechanical properties of BF and BFRP caused the localized fibre breakage to occur. In the SEM image of the CFRP-T800 tube, a small amount of fibres was pulled out with partial resin adhesion, but the size of the pulled-out fibres was small. Similarly to the case of the CFRP-T800 tube, only a few fibres were pulled out in the case of the CFRP-T700 tube, and a neat and packed cross-section could be observed. Relating to the mechanical properties, it can be understood that the failure mechanism of the two is similar when subjected to impact. In particular, in the SEM image of GFRP tube, partial glass fibres were pulled out from the resin because of their large elongation; whereas a large amount of resin adhered to the pulled-out fibres, indicating that the adhesion between the glass fibre and resin matrix was strong and the glass fibres were completely incorporated into the resin. Therefore, the GFRP tube shows the best impact resistance, which related to the infiltration of fibres in resins, and the toughness of the fibres.

**Figure 11:**
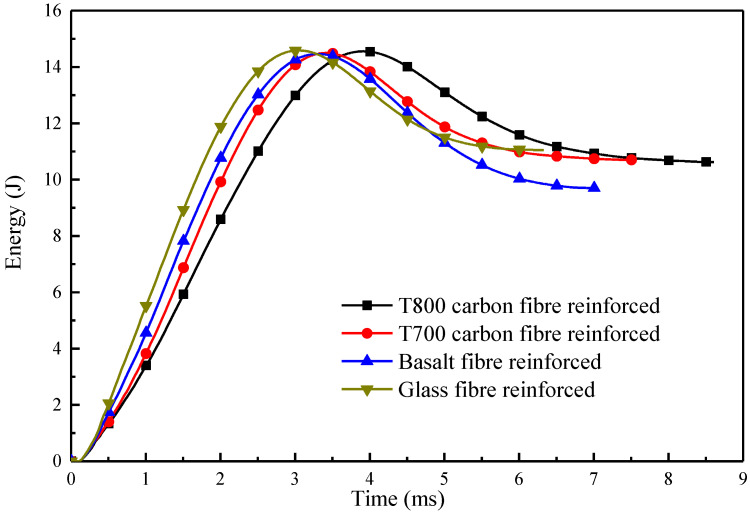
Energy–time (E–T) diagrams for four types of fibre-reinforced composite tubes.

**Figure 12:**
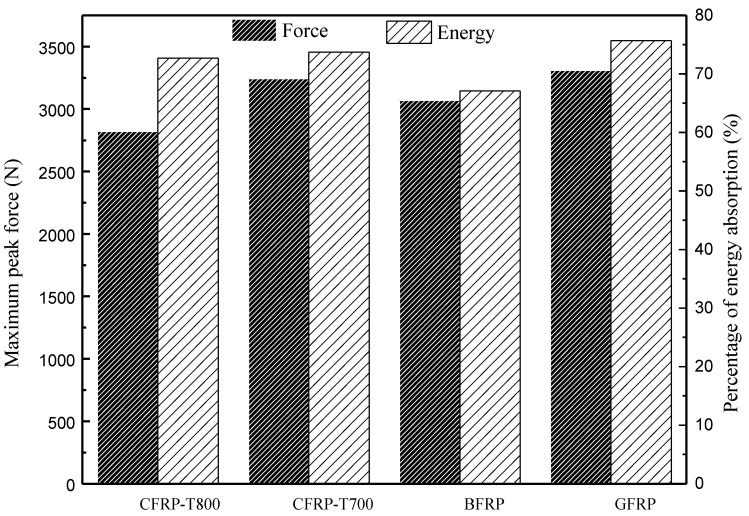
Maximum peak forces and percentage of energy absorption of four types of fibre-reinforced composite tubes.

Figure 11 represents the energy–time (E–T) diagrams of the four types of fibre-reinforced composite tubes during the impact process. The internal energy in the tubes first increase and subsequently decrease to a stable value. The maximum internal energy of the four tubes reinforced by CFRP-T800, CFRP-T700, BFRP and GFRP are 14.56 J, 14.49 J, 14.46 J and 14.59 J, respectively; as soon as the internal energy reaches a maximum value, the impactor starts to rebound. The initial impact energy set in the experiment is 15 J. The maximum internal energies of the four tubes obtained by the impact sensor are about 14.5 J, and the error is within the acceptable range, implying that the impact sensor is accurate. The final energies in the tubes are 10.62 J, 10.69 J, 9.7 J and 11.05 J, respectively, which are the absorbed energies of the tubes. To compare the impact damage behaviour, the percentage of energy absorption (*r*) is calculated as

(2) $$r=\frac{E_{a}}{E_{i}}\times 100\%$$

where *E_a_* is the absorbed energy and *E_i_* is the maximum internal energy. The calculated values of r of the four tubes are 72.7%, 73.7%, 67.1% and 75.7%, respectively. Figure 12 clearly shows that the GFRP tube has the maximum peak force as well as the highest value of *r*, which indicates that the strong interfacial adhesion between the fibres and resin, and the elongation of the glass fibres improves the structural impact resistance of the tubes. The value of *r* of the CFRP-T800 and CFRP-T700 tubes are close, which is related to the similarity of the previous failure mechanism. Fibre breakage occurs globally around the impact centre and thus absorbs more energy. The GFRP tube absorbs plenty of energy through delamination and deformation, of which the residual deformation ratio of the GFRP tube reaches the most (Figure 8). In contrast, the value of r of the BFRP tube is much lower than the other three tubes, indicating that the BFRP tube showed the lowest structural impact resistance. It can be seen from Figure 9c that the BFRP tube mainly realizes energy absorption through delamination damage. Although localized fibre breakage occurs, the energy absorbed by delamination is much smaller [25]. Therefore, the BFRP tube absorbed the least energy. Relevant information about the impact behaviour of all composites is summarized in Table 3.

### 3.3. Radial Compression after Impact Response and Behaviour

**Figure 13:**
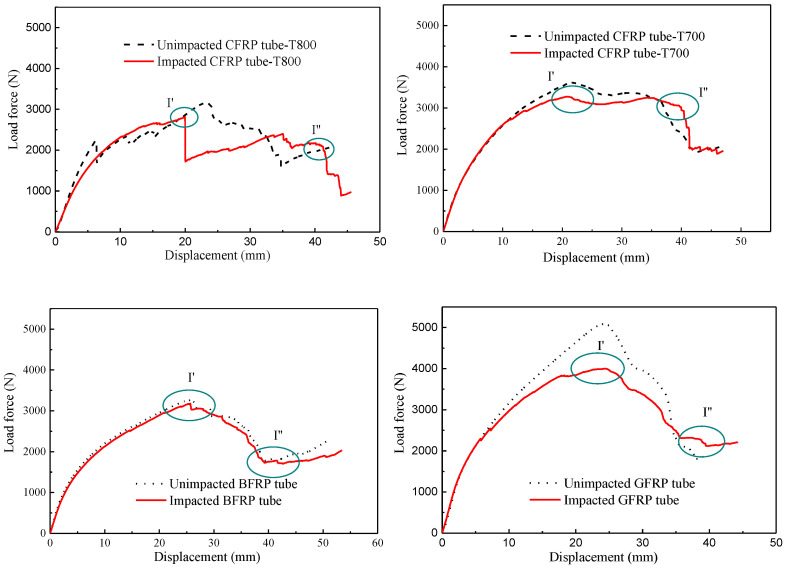
Typical compression load–displacement curve.

Radial residual compression strength (R-RCS) tests were conducted to investigate the loss of compressive strength of the specimen. Figure 13 shows the compression load–displacement curve for the four types of impacted and un-impacted fibre-reinforced tubes, including CFRP-T800, CFRP-T700, BFRP and GFRP, during R-RCS tests. The curves show that the LVI behaviour has different effects on the residual compression performance of the four fibre-reinforced tubes. To compare the impact damage behaviour of these four types of fibre-reinforced composite tubes, the percentages of R-RCS ratios (*R*) [26,27] can be calculated as

(3) $$R\left(\%\right)=\frac{F_{max,\;impacted}}{F_{max,unimpacted}}\times 100\%$$

where *F_max_* is R-RCS peak load. The values of *R* of the CFRP-T800, CFRP-T700, GFRP and BFRP tubes of the fibre-reinforced tubes are 88.1%, 90.7%, 97.3%, and 73.8%, respectively. The R-RCS of the impacted BFRP tube showed little variation compared to the un-impacted tube. This is related to the fact that the BFRP tube absorbed less energy. As the failure mechanism and absorption energy of CFRP-T800 and CFRP-T700 tube differ little under LVI, their retention rates of compression performance after impact are relatively close. Due to the occurrence of global fibre breakage, the compression performance after impact decreases significantly. It should be noted that during the compression process of the impacted CFRP-T800 tube, a severe load drop appears at the position of I’, indicating that the LVI has a great impact on the radial compression performance of the CFRP-T800 tube. On the contrary, the GFRP tube absorbed more energy leading to a lower R-RCS. Among the four types of tubes, the R-RCS of the BFRP tube is not sensitive to impact and that of the GFRP tube is highly sensitive to impact.

**Figure 14:**
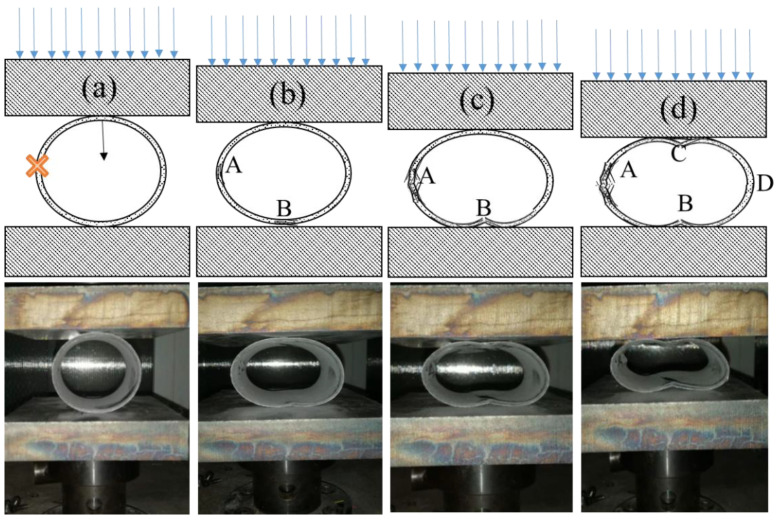
Images of CFRP-T700 tubes under radial compression. (**a**) uncompressed state, (**b**) damages start, (**c**) damages extension and (**d**) collapse of the tube.

To compare the damage evolution during the R-RCS test of the tubes with different fibres, images of the composite tubes under radial compression are shown in Figure 14. There appears to be elastic deformation at the initial stage of the compression process, as shown in Figure 14. The compression process of four types of FRP tubes differs little, taking the CFRP-T700 tube as an example. With deepening of the deformation, the compression load reaches the maximum value and thereafter begins to fall drastically. Evident delamination appears at the impact position A in image (b), indicating that the strength of tubes decreased and thus caused damage. Fibre fracture and warp up at position B in image (c) can be attributed to the bending stress concentration in the central face under the compression stage; position I’ corresponds to this in Figure 13. In Figure 14d, the upper and lower cambered surfaces are clearly bent and damaged; position I” in Figure 13 corresponds to the damage on the opposite side of the impact centre. After the compression displacement exceeds (d), the degree of damage of the tube increases and the force drops continuously until the structures on both sides are completely destroyed; after the upper and lower arch surfaces are fully stressed, the force begins to increase once again. According to the order of damage, damage occurs first in impact position during compression. Therefore, LVI has a considerable influence on the R-RCS of composite tubes.

## 4. Conclusions

This study investigates the effect of fibres on the LVI behaviour, failure mechanism, and RCS of tubular composite structures reinforced by carbon fibre-T800, carbon fibre-T700, basalt fibre, and glass fibre. The following conclusions can be drawn from the results obtained.

- Delamination and fibre breakage damage are the main failure mechanisms of FRP tubes when subjected to LVI. The damage degree depends on the interfacial adhesion between the fibres and resins, and the mechanical properties of the tubes. Global fibre breakage damage occurs in the CFRP-T800 and CFRP-T700 tubes, while localized fibre breakage occurs in the BFRP and GFRP tubes.
- The residual deformation ratios of the composite tubes show the following trend: GFRP tube > CFRP tubes > BFRP tube—the same as the percentages of energy absorption. The residual deformation ratio and the energy absorption percentage of CFRP-T700 and CFRP-T800 tubes differ little, which related to their mechanical properties. The GFRP tube absorbs the most energy through delamination and large deformation, which shows the best impact resistance. The BFRP tube absorbs the lowest energy, mainly by delamination, which shows the poorest impact resistance.
- LVI has a considerable influence on the R-RCS of composite tubes. The retention rates of R-RSC of the four tubes are 88.1%, 90.7%, 97.3%, and 73.8%. The BFRP tube is not sensitive to impact, and the GFRP tube is highly sensitive to impact.

## Figures and Tables

**Table 1:** Mechanical properties of four types of fibres.

Types	Tensile Strength (MPa)	Tensile Modulus (GPa)	Elongation at Break (%)
Carbon fibre-T800	5880	294	1.9
Carbon fibre-T700	4900	230	2.1
Basalt fibre	3500	100	3.2
Glass fibre	2800	86	4.8

**Table 2:** Material parameters.

Materials	Tensile Strength/MPa	Tensile Modulus/GPa	Poisson’s Ratio	Shear Strength/MPa	Density/g·cm^3^
CFRP-T800	1336.1 (1)	137.9 (1)	0.26 (12)	56.4 (12)	1.43
CFRP-T700	1263.2 (1)	128.7 (1)	0.27 (12)	57.01 (12)	1.61
BFRP	648.3 (1)	50.2 (1)	0.30 (12)	55.64 (12)	1.93
GFRP	793.2 (1)	43.7 (1)	0.28 (12)	57.30 (12)	1.90

**Table 3:** Relevant parameters about impact behaviour of all composite tubes.

Types	Failure Mechanism	Residual Deformation Ratio (%)	*r* (Percentage of Energy Absorption, %)
CFRP-T800	Global fibre breakage and delamination damage	20.4	72.7
CFRP-T700	Global fibre breakage and delamination damage	20.5	73.7
BFRP	Localized fibre breakage and delamination damage	20.0	67.1
GFRP	Localized fibre breakage and delamination damage	32.0	75.7
